# Microscopic Visualisation of Zoonotic Arbovirus Replication in Tick Cell and Organ Cultures Using Semliki Forest Virus Reporter Systems

**DOI:** 10.3390/vetsci3040028

**Published:** 2016-09-29

**Authors:** Lesley Bell-Sakyi, Sabine Weisheit, Claudia Rückert, Gerald Barry, John Fazakerley, Rennos Fragkoudis

**Affiliations:** 1 The Pirbright Institute, Ash Road, Pirbright, Surrey GU24 0NF, UK; Sabine.Weisheit@rr-research.no (S.W.); claudia.rueckert@colostate.edu (C.R.); john.fazakerley@unimelb.edu.au (J.F.); rennos.fragkoudis@pirbright.ac.uk (R.F.); 2 The Roslin Institute and Royal (Dick) School of Veterinary Studies, University of Edinburgh, Easter Bush, Midlothian EH25 9RG, UK; gerald.barry@ucd.ie

**Keywords:** tick cell line, tick organ culture, arbovirus, Semliki Forest virus, reporter gene, microscopy

## Abstract

Ticks are vectors and reservoirs of many arboviruses pathogenic for humans or domestic animals; in addition, during bloodfeeding they can acquire and harbour pathogenic arboviruses normally transmitted by other arthropods such as mosquitoes. Tick cell and organ cultures provide convenient tools for propagation and study of arboviruses, both tick-borne and insect-borne, enabling elucidation of virus-tick cell interaction and yielding insight into the mechanisms behind vector competence and reservoir potential for different arbovirus species. The mosquito-borne zoonotic alphavirus Semliki Forest virus (SFV), which replicates well in tick cells, has been isolated from *Rhipicephalus*, *Hyalomma*, and *Amblyomma* spp. ticks removed from mammalian hosts in East Africa; however nothing is known about any possible role of ticks in SFV epidemiology. Here we present a light and electron microscopic study of SFV infecting cell lines and organ cultures derived from African *Rhipicephalus* spp. ticks. As well as demonstrating the applicability of these culture systems for studying virus-vector interactions, we provide preliminary evidence to support the hypothesis that SFV is not normally transmitted by ticks because the virus does not infect midgut cells.

## 1. Introduction

Ixodid and argasid ticks transmit a wide range of pathogenic arboviruses of medical and/or veterinary importance; most of these are RNA viruses belonging to the families Bunyaviridae, Flaviviridae, and Reoviridae, while a single DNA virus, African swine fever virus of the family Asfarviridae, is harboured and transmitted by argasid ticks of the genus *Ornithodoros* [1]. Ticks also harbour many viruses that are not known to cause disease in humans or livestock, but can replicate in vertebrate cells [2]. Recently, the existence of a third group of apparently endogenous “tick-only” viruses has been recognised; of these only one, the orbivirus St Croix River virus, has been fully sequenced and characterised in vitro [3,4]. Other putative endogenous tick viruses have only been detected by electron microscopy in cultured tick cells [4,5,6], as partial RNA sequences identified through high-throughput screening of field tick samples (reviewed by [7]), or as partial DNA sequences present in tick genomes [8]. A fourth group of viruses detected in ticks by in vitro isolation and/or molecular techniques comprises arboviruses that are normally transmitted by other arthropods, in particular mosquitoes. These include zoonotic pathogens such as West Nile virus, Rift Valley fever virus, and chikungunya virus [9,10,11].

Continuous tick cell lines have been used for propagation and study of tick-borne and insect-borne arboviruses for over 40 years (reviewed by [12]). Many of the arboviruses harboured by ticks can be propagated in tick cell cultures, whether or not ticks are their usual vectors [12]. One such virus, the mosquito-borne *Alphavirus* Semliki Forest virus (SFV), has proved to be a useful model arbovirus for a range of different in vitro studies in tick cells [13,14,15,16,17]. In vitro, SFV infects and replicates in arthropod cell lines of both vector (mosquito) and non-vector (tick) origin, as determined by titration on mammalian cells and using virus constructs incorporating reporter genes for fluorescent or luminescent proteins [16,18,19,20]. By electron microscopy (EM), structures associated with SFV replication complexes have been described in infected mammalian and mosquito cells; these include two types of cytopathic vacuole (CPV): CPV-I in which membranous spherules project from the interior surface, and CPV-II on which viral nucleoids are arranged in arrays on the surface of areas of dilated endoplasmic reticulum [21,22,23]. However, SFV replication and production of virus particles in tick cells has not been visualised by EM, and moreover there are few EM studies illustrating actual tick-borne viruses in tick cell lines [4,5,6,24,25].

Tick organ cultures also provide useful model systems for studying tick-microorganism interactions at the tissue/organ/whole organism level. Developing adult explant cultures [26] were used for propagation of Colorado tick fever virus [27], long-term infection with tick-borne encephalitis virus (TBEV) and Powassan virus [28,29], and in vitro development of the protozoan parasite *Theileria annulata* [30]. Such organ culture systems offer potential for wider application, especially with the advent of reporter viruses capable of expressing fluorescent proteins that can be used for rapid light microscopic identification of infected cells and tissues [31,32,33].

In the present study we used different approaches to demonstrate the application in arbovirus research of tick cell and organ cultures combined with reporter viruses. We used light microscopy and EM to visualise SFV replication complexes and mature virus particles in a *Rhipicephalus decoloratus* cell line. We included *Aedes albopictus* mosquito and mammalian cells infected with SFV as positive controls for the infected tick cells. We used light microscopy to examine the distribution of SFV-infected tissues in *Rhipicephalus evertsi* developing adult organ cultures and to determine whether or not cells with midgut morphology in a *R. evertsi* cell line become infected with SFV.

## 2. Materials and Methods

### 2.1. Cell Lines

The *R. decoloratus* tick cell line BDE/CTVM16 [34] at passage level 76 was grown in L-15 (Leibovitz) medium supplemented with 10% tryptose phosphate broth (TPB, Invitrogen, Paisley, UK), 20% foetal bovine serum (FBS, Labtech International, Uckfield, UK), 2 mM L-glutamine, and antibiotics (100 units/mL penicillin, and 100 µg/mL streptomycin; Sigma-Aldrich, Poole, UK) (complete L-15) at 28 °C. The *R. evertsi* tick cell line REE/CTVM28 [6] at passage level 36 was grown in a 1:1 mixture of L-15 (Leibovitz) medium and Minimal Essential Medium (MEM) with Hanks’ salts (Sigma-Aldrich, Poole, UK), supplemented as above, at 28 °C. Prior to virus infection, tick cells were seeded in 2 mL volumes in sealed flat-sided culture tubes (Nunc) at a density of 8 × 10^5^ cells/mL and incubated overnight. The *Ae. albopictus* mosquito cell line C6/36 [35] was grown in L-15 (Leibovitz) medium supplemented with 10% TPB and 10% FBS [36] at 28 °C. The mammalian cell line BHK-21 [37] was grown in Glasgow MEM medium (Invitrogen, Paisley, UK supplemented with 10% newborn calf serum (Seralab, West Sussex, UK), 10% TPB, and antibiotics as above, at 37 °C in a humidified atmosphere of 5% CO_2_ in air. Prior to virus infection, C6/36 and BHK-21 cells were seeded in 2 mL volumes in six-well plates at densities of, respectively, 8 or 3 × 10^5^ cells/well and incubated overnight.

### 2.2. Tick Organ Cultures

Engorged nymphal *R. evertsi* ticks, kindly provided by Ard Nijhof and Frans Jongejan, Utrecht Centre for Tick-borne Diseases, were processed as described previously [30] about half way through the pre-eclosion period when developing adult tissues were visible at the anterior of the nymphal integument. Briefly, moulting nymphs were surface-sterilised in 0.1% benzalkonium chloride (5 min) and 70% ethanol (1 min), dried, embedded dorsal side uppermost in sterile wax and immersed in Hanks balanced salt solution. The dorsal half of the nymphal integument was removed by cutting round the midline and severing the tracheae at the spiracles, then each “whole nymph explant” was placed on a sterile glass 6 × 22 mm coverslip with 0.2 mL of complete L-15 medium inside a sealed flat-sided culture tube and incubated at 28 °C overnight prior to virus infection.

### 2.3. Viruses

The SFV construct SFV4(3F)-ZsGreen [32], expressing ZsGreen fused to the non-structural protein nsP3 and resulting in punctate green fluorescence associated with viral replication complexes, was used to infect the cell cultures. This enabled quantification by light (fluorescence) microscopy both of infected cells per culture and of replication complexes per cell, prior to harvest and fixation for EM. Following overnight incubation, seeded BDE/CTVM16, C6/36, BHK-21, and REE/CTVM28 cells were infected with SFV4(3F)-ZsGreen suspended in phosphate buffered saline (PBS) with 0.75% bovine serum albumin (PBSA) at a multiplicity of infection of 10 to ensure simultaneous infection of maximum numbers of cells in each culture. The SFV construct SFV4-steGFP [31], expressing enhanced green fluorescent protein (eGFP) as a cleavable component of the structural open reading frame which results in diffuse green fluorescence within virus-infected arthropod cells, was used to infect *R. evertsi* organ cultures by directly adding virus suspended in PBSA.

### 2.4. Monitoring of Virus-Infected Cultures

SFV-infected BDE/CTVM16, C6/36, and BHK-21 cell cultures were examined by fluorescence microscopy (Axio Observer inverted microscope, Carl Zeiss, Jena, Germany) to determine the percentage of infected cells and the approximate number of replication complexes within the infected cells. SFV-infected REE/CTVM28 cell cultures were examined as above to investigate whether midgut-like cells were infected with virus. SFV-infected and uninfected *R. evertsi* whole nymph explants were examined by light and fluorescence stereoscopic microscopy (Stemi SV 11 binocular dissecting microscope, Carl Zeiss, Jena, Germany), and inverted microscopy as above, to determine location of infected tissues. Photographs were taken with a charge-coupled device (CCD) digital camera and analysed with Axiovision software (Carl Zeiss, Jena, Germany).

### 2.5. Harvest, Fixation, and Processing of Cells for EM

One sample was collected from each of the uninfected (mock-infected) cell lines, and two samples, representing early and late stages of SFV infection, were collected from each of the infected cell lines. These time points are detailed in Table 1. Cells were harvested by gentle detachment and centrifuged at 200× *g* for 5 min; the supernatant medium was discarded, the cell pellet was resuspended gently in 1 mL PBS and centrifuged as before. The supernatant PBS was discarded and the cell pellet was fixed and processed as described previously [6]. Sections were viewed in a Phillips CM120 transmission electron microscope. Images were taken using a Gatan Orius CCD camera.

## 3. Results

### 3.1. Ultrastructural Visualisation of SFV in Infected Cells

Structures associated with SFV replication and production were clearly visible by EM in tick, mosquito, and mammalian cells infected with SFV4(3F)-ZsGreen. These included structures with features resembling those of CPV-I and CPV-II associated with virus replication in mammalian and mosquito cells [21,22,23,38], and apparently mature virus particles being released from the outer cell membrane. No structures resembling any virus particles or replication complexes were seen in mock-infected control cells (data not shown).

#### 3.1.1. SFV in Tick Cell Line BDE/CTVM16

**Figure 1:**
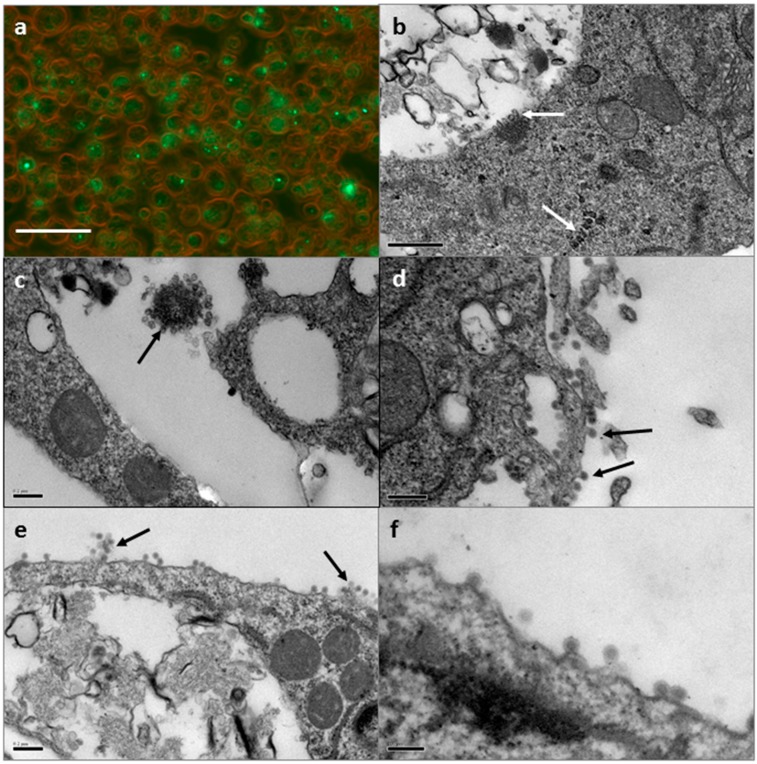
SFV in the tick cell line BDE/CTVM16. (**a**) Light micrograph of SFV-infected tick cells at 48 hpi; foci of green fluorescence correspond to location of viral replication complexes. Scale bar 50 µm; combined brightfield and ultra-violet (UV) illumination; (**b**–**f**) Electron micrographs of SFV-infected tick cells at 24 hpi; (**b**) Array of intracellular spherules resembling those associated with CPV-I (arrows); scale bar 0.5 µm; (**c**) Cluster of intracellular spherules (arrow); scale bar 0.2 µm; (**d**,**e**) Virus particles (arrows) at the surface of the cell membrane; scale bar 0.2 µm; (**f**) Higher magnification view of virus particles at the cell surface; scale bar 0.1 µm.

By light microscopy, 93% of BDE/CTVM16 cells were detectably infected with SFV at 24 h post infection (hpi), with 1–9 visible foci of fluorescence (equivalent to replication complexes) per cell (data not shown). At 48 hpi foci of fluorescence were seen in 81% of cells, with 1–6 foci per cell (Figure 1a). At 24 hpi, EM examination revealed small arrays of spherule-like structures in the cytoplasm resembling those lining CPV-I (Figure 1b) and a cluster of spherule-like structures within a large intracellular vacuole (Figure 1c); however, these were not as distinct and regularly arranged as those described previously [21,22,38]. Numerous alphavirus-like particles were seen budding or being released from the outer membrane of some of the cells at 24 (Figure 1d–f) and 48 hpi. No structures resembling CPV-II in mammalian cells were seen by EM in BDE/CTVM16 cells at either time point.

#### 3.1.2. SFV in Mosquito Cell Line C6/36

**Figure 2:**
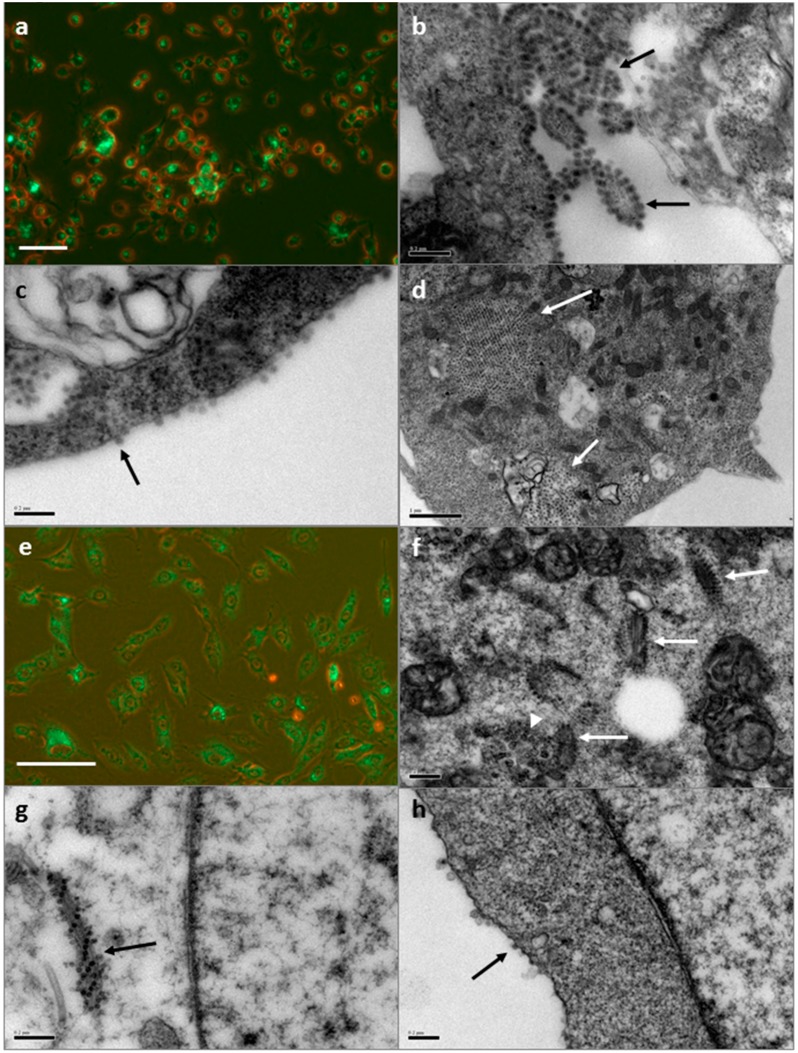
SFV in the mosquito cell line C6/36 and mammalian cell line BHK-21. (**a**) Light micrograph of SFV-infected C6/36 cells at 10 hpi; foci of green fluorescence correspond to location of viral replication complexes. Scale bar 50 µm; combined brightfield and UV illumination; (**b**,**c**) Electron micrographs of SFV-infected C6/36 cells at 24 hpi; (**b**) Arrays of nucleiods associated with putative CPV-II (arrows); scale bar 0.2 µm; (**c**) Putative virus particles (arrow) at the surface of the cell membrane; scale bar 0.2 µm; (**d**) Endogenous virus in uninfected C6/36 cell (arrows); scale bar 1 µm; (**e**) Light micrograph of SFV-infected BHK-21 cells at 10 hpi; foci of green fluorescence correspond to location of viral replication complexes; scale bar 50 µm; combined brightfield and UV illumination; (**f**–**h**) Electron micrographs of SFV-infected BHK-21 cells; scale bars 0.2 µm; (**f**) Spherules associated with putative CPV-I (arrowhead) and numerous nucleoids associated with putative CPV-II (arrows) at 6 hpi; (**g**) CPV-II (arrow) at 6 hpi; (**h**) Putative spherules and virus particles at the cell surface (arrow) at 10 hpi.

By light microscopy, the detectable infection rate of C6/36 cells with SFV at 10 hpi was 98%, with 1–15 visible foci of fluorescence per cell (Figure 2a). A similar picture was seen at 24 hpi. In C6/36 cells at 10 hpi, structures similar to those previously labelled as CPV-II [22] (Figure 2b) and mature virions associated with membranes and budding from the cell surface were seen by EM. At 24 hpi, numerous apparently mature virus particles were seen at the outer membrane of infected cells (Figure 2c). Structures convincingly resembling CPV-I were not seen by EM in C6/36 cells at either time point. However, both SFV-infected and mock-infected C6/36 cells were found to harbour large accumulations of icosahedral virus-like particles with a diameter of 55–60 nm both within intracytoplasmic vacuoles and apparently free in the cytoplasm, sometimes forming crystalline arrays (Figure 2d). The identity of this apparently endogenous virus is unknown.

#### 3.1.3. SFV in BHK-21 Cells

By light microscopy, 97% of BHK-21 cells were detectably infected with SFV at 6 hpi, with around 10–50 visible foci of fluorescence per cell (data not shown). By 10 hpi 100% of cells were infected (Figure 2e). By EM, spherule-like structures resembling those associated with CPV-I (Figure 2f) and nucleoid/nucleocapsid-like structures resembling those associated with CPV-II [21,22] (Figure 2f,g) were seen at both time points; the latter appeared to be arranged in spirals or all over the surface of small sections of host membranes. At 10 hpi, many of the CPV-II-like structures seen at 10 hpi were present and putative external spherules [39] and virus particles apparently budding or being released from the cell surface were seen (Figure 2h).

### 3.2. Distribution and Cell Tropism of SFV in Infected Tick Organ and Cell Cultures

#### 3.2.1. SFV in *R. evertsi* Organ Cultures

**Figure 3:**
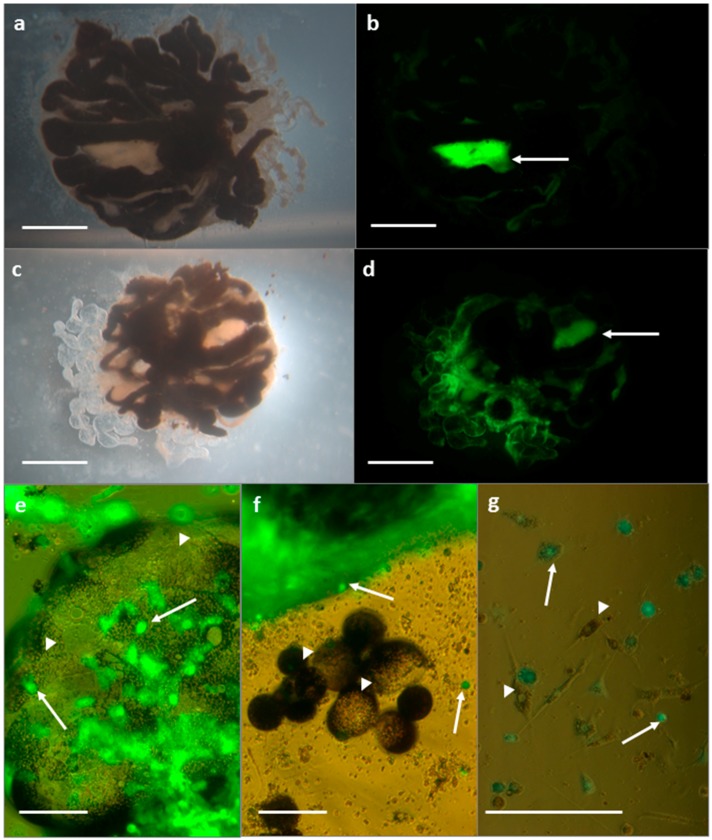
*Rhipicephalus evertsi* developing adult explants 48 hpi viewed under light (**a**,**c**), UV (**b**,**d**), and combined (**e**–**g**) illumination. **(a**,**b**) Uninfected control explant; arrow indicates autofluorescence of guanine crystals in the rectal sac; scale bars 1 mm; (**c**,**d**) SFV-infected explant; eGFP fluorescence indicates SFV infection in developing adult tissues; arrow indicates autofluorescence of guanine crystals in the rectal sac; scale bars 1 mm; (**e**–**g**) SFV-infected explant with green fluorescence indicating virus-infected developing adult tissues and haemocytes (arrows) and absence of fluorescence in midgut cells (arrowheads); scale bars 100 µm.

Uninfected developing adult organ cultures displayed strong autofluorescence of the white guanine crystals contained within the rectal sac (Figure 3a,b) while other tissues, or haemocytes that had migrated or floated outwards from the main explant, did not fluoresce. At 48 hpi, cultures infected with SFV4-steGFP additionally showed specific eGFP fluorescence in light-coloured tissues of the developing adult limbs, mouthparts, integument, and fat body, but the black midguts did not fluoresce (Figure 3c,d). Some of the explant cultures contained individual midgut cells and haemocytes around the periphery of the explants; in SFV-infected cultures, midgut cells, identified by their black or dark brown cytoplasmic inclusions, did not show any fluorescence, while developing adult tissues and some, but not all, haemocytes fluoresced strongly (Figure 3e–g).

#### 3.2.2. SFV in *R. evertsi* Cell Line REE/CTVM28

**Figure 4:**
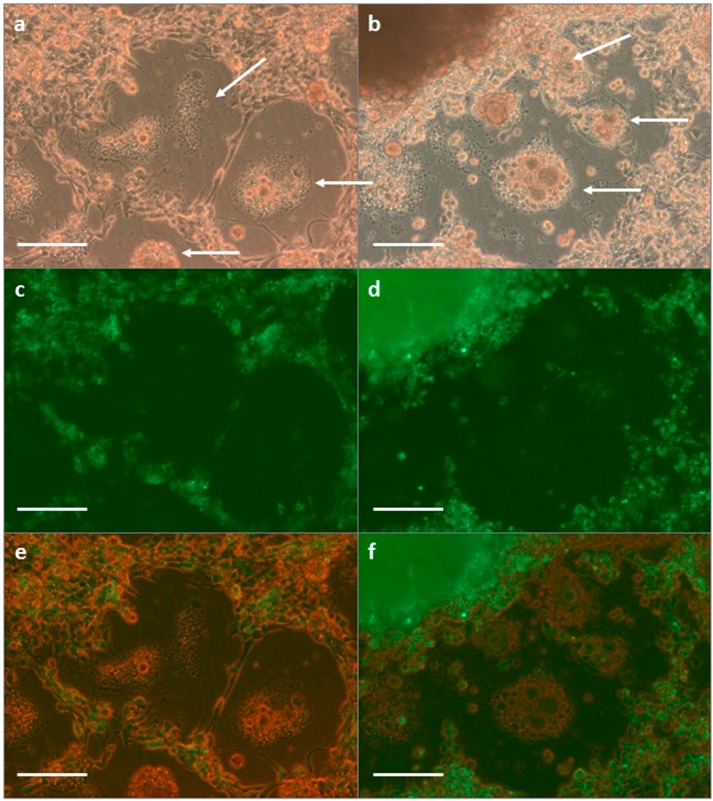
SFV in the tick cell line REE/CTVM28 at 48 HPI, viewed live under brightfield (**a**,**b**); UV (**c**,**d**); and combined (**e**,**f**) illumination, scale bars 100 µm. Two fields are shown (**a**,**c**,**f**; **b**,**d**,**h**) illustrating presence of punctate green fluorescence indicating SFV infection in many small cells, but absence of fluorescence in large foamy cells with midgut morphology (arrows).

To further examine the observation that the midgut epithelial cells in the *R. evertsi* developing adult explant cultures did not appear to be infected with SFV, based on the absence of fluorescence from these tissues, the *R. evertsi* cell line REE/CTVM28 was infected with SFV4(3F)-ZsGreen and examined by light microscopy (Figure 4). REE/CTVM28 contains a mixture of cell phenotypes, including large, flattened vacuolated cells (Figure 4a,b) that resemble midgut cells seen migrating out from developing adult explants. At 48 hpi, foci of fluorescence corresponding to SFV replication complexes could be clearly seen in many cells in the infected cultures, with the exception of cells with midgut morphology (Figure 4c–f). Foci of green fluorescence were not present in any cells of mock-infected REE/CTVM28 cultures.

## 4. Discussion

Using a model arbovirus, SFV, we have shown here that tick cell lines and organ cultures have considerable potential as model systems to examine the interactions between arboviruses and ticks at the cellular, tissue, and whole organism level. Our study demonstrates the importance of careful selection of lines appropriate for the virus and purpose from amongst the wide range of tick cell lines now available [12,40]. We used the *R. decoloratus* cell line BDE/CTVM16 because we had previously shown that this cell line supports replication of SFV to relatively high titres as determined by luciferase assay in comparison to other tick cell lines [16], making it particularly suitable for an EM study in which a high infection rate is important. The *R. evertsi* cell line REE/CTVM28, also previously shown to support replication of SFV [16], contains, amongst others, cells that resemble those of midgut origin (unpublished data), making it particularly suitable for investigating whether or not SFV can infect tick midgut cells in conjunction with the *R. evertsi* organ cultures. It would be interesting to repeat the experiments described here with a tick-borne virus expressing a fluorescent reporter gene. Green fluorescent protein-expressing TBEV replicons reported previously [41,42] could be used at biosafety level (BSL) 2 to monitor initial infection of cells but not virus spread, whereas the eGFP-expressing infectious TBEV vector reported previously [42] would require handling at BSL 3. We did not have access to any of these TBEV constructs at the time when the *R. evertsi* developing adult ticks were available.

While tick cell lines have been known to provide a suitable substrate for propagation and study of arboviruses for nearly half a century [12], there have been surprisingly few published morphological studies depicting actual arboviruses in tick cells in vitro. *Rhipicephalus appendiculatus* RA243 cells infected with the tick-borne nairovirus Nairobi sheep disease virus (NSDV) were examined by EM [5]; occasional small groups of bunyavirus particles were seen in the cell cytoplasm and also in the intracellular space. These authors also reported the presence in RA243 cells of *Reovirus*-like particles that were more numerous and more frequently observed in NSDV-infected cultures; these were also illustrated in the paper.

Using a technique similar to that of the present study, infected developing adult *Hyalomma anatolicum* and *Hyalomma dromedarii* explant cultures were infected with two tick-borne flaviviruses, TBEV, and Powassan virus [28]. Using EM, these authors were able to visualise morphologically mature virus particles intracellularly in expanded endoplasmic reticulum systernae and also rarely in intracellular spaces. In later stages of persistent infection, many incomplete virus particles lacking an electron-dense centre, as well as crystalline arrays of particles, were seen; photomicrographs of TBEV-infected cells at days 15 and 60 post infection are shown [28]. In two studies, the ultrastructural morphology and maturation process of TBEV in the cytoplasm of cells of the *R. appendiculatus* cell line RA257 were described [24,25]. Virions were observed and illustrated 3–4 days post infection both inside large cytoplasmic vacuoles and free in the cytoplasm; the latter were often in close proximity to smaller nucleocapsid-like particles. Virus egress from the cells was not reported. Photomicrographs of putative endogenous viruses in several ixodid and argasid tick cell lines have been published [4,6]; the identity of these viruses remains unknown.

Tick (RA243) cells infected with the tick-borne nairovirus Dugbe virus were examined by EM [43]; the single photomicrograph of an infected RA243 cell showed a viral inclusion body but no virus particles. In a recent ultrastructural study of infection of cells of the *Ixodes scapularis* tick cell line ISE6 with another tick-borne flavivirus, Langat virus, round vesicles and tubular structures of unknown function were associated with endoplasmic reticulum in, respectively, acute and persistent infection, but mature virus particles were rarely seen in ISE6 cells and were not illustrated in the paper [44].

None of the aforementioned studies demonstrated release of mature virus particles from the surface of infected tick cells. Here we have shown for the first time the appearance of an arbovirus, SFV, budding or being released from the surface of a tick cell. We also detected structures associated with SFV replication similar to those reported to occur in mammalian and mosquito cells. The ultrastructure of SFV replicating in primary chick embryo cells and in the mammalian cell line BHK-21 was reported by previous workers [21,22,38], who described three distinct morphological stages: CPV-I, CPV-II, and mature virions budding from the cell surface. The first stage, CPV-I, was detected at 1–2.5 hpi; CPV-II appeared at 4 hpi and mature virions were seen from 2.5 hpi. In the present study we detected numerous structures resembling CPV-II in BHK-21 cells [22] at both 6 and 10 hpi, and mature virions at 10 hpi. In contrast, we only found a single example of spherules associated with a putative CPV-I, possibly due to our first BHK-21 samples being taken at 6 hpi when the numbers of CPV-I would be expected to be declining [21]. Using a correlative light and EM technique, a CPV-I-like structure was detected in SFV-infected C6/36 cells at 20 hpi [23], while we saw structures resembling CPV-II [22] and mature virions outside the cell membrane at 10 and 24 hpi, but no CPV-I. In contrast to the mosquito cells, we did not see any structures resembling CPV-II in BDE/CTVM16 cells, but detected spherule-like structures possibly derived from putative CPV-I at 24 hpi; however, these were not as distinct and regularly arranged as those described previously [21,22]. It is possible that, by examining earlier time points of infection in both BHK-21 cells and arthropod cells, we would have gained a clearer and more comprehensive picture of SFV replication and production in the different host cells, including observation of early-stage spherules at the plasma membrane [39]. However, the main purpose of this study was to demonstrate the applicability of the culture systems to virus infection of tick cells, for which we considered that two time points, representing early and late stages of infection in each cell type [13,19,32], were sufficient.

Although alphaviruses have occasionally been isolated from field ticks removed from mammalian hosts [45,46], and experimental transstadial transmission of Venezuelan equine encephalitis virus between guinea pigs by the ticks *Amblyomma cajennense* and *Hyalomma truncatum* has been demonstrated [47,48], ticks are not normally considered to be vectors of alphaviruses in the field. Possible explanations for this may be that alphaviruses naturally ingested in the bloodmeal during feeding are normally unable either to infect midgut cells or to penetrate the midgut escape barrier (MEB) and cause systemic infection [2]. To investigate this, we used developing adult organ cultures in which midgut and other tissues retain their structure and relative distribution, and a tick cell line in which cells with both midgut and non-midgut morphology are present. In both cases, non-midgut cells became infected with SFV reporter viruses as indicated by the presence of green fluorescence, while midgut cells whether in intact organs or as individual cells, did not. Our results, although not conclusive, indicate that SFV cannot infect *R. evertsi* midgut cells, rather than that midgut cells become infected but the virus cannot penetrate the MEB. It is possible that, since the SFV4-steGFP construct used to infect the explants expresses eGFP late in the replication process with the structural proteins, the virus could have entered the explant midgut cells and initiated replication of the non-structural proteins but had been unable to proceed further. However, absence of green fluorescence in REE/CTVM28 cells with midgut morphology following infection with SFV4(3F)-ZsGreen, which expresses ZsGreen early in the replication process with the non-structural proteins, does not support this view. It is also possible that failure of SFV to replicate in *R. evertsi* midgut cells in the present study, in which the route of infection was not by natural bloodfeeding on an infected host, was due to absence in vitro of unknown factors associated with the uptake of a bloodmeal. Further studies using ticks infected with reporter viruses naturally via the oral route are needed to determine conclusively whether or not SFV in the bloodmeal can infect and replicate in midgut cells in vivo and whether or not it can penetrate the MEB. Once past the MEB, our results suggest that systemic infection of tick tissues would be possible, though we did not demonstrate infection of salivary glands and thereby likely ability to transmit the virus during feeding.

## 5. Conclusions

In conclusion, the combination of appropriate vector and non-vector tick cell and organ culture systems with viruses expressing reporter genes, as illustrated in the present study, offers enormous potential for future exploration of tick cell and tissue interactions with a wide range of arbovirus groups for which reverse genetics techniques are increasingly available. Such studies will undoubtedly help to elucidate the factors underlying vector competence and possible reservoir capabilities.

## Figures and Tables

**Table 1:** Sample time points for each cell line examined by electron microscopy. Each sample represented the entire contents of a single well or tube of cells; hpi = hours post infection with Semliki Forest virus (SFV) or mock infection.

Cell Line		First Sample	Second Sample
BDE/CTVM16	Mock-infected	24 hpi	48 hpi
	SFV-infected	24 hpi	
C6/36	Mock-infected	24 hpi	24 hpi
	SFV-infected	10 hpi	
BHK-21	Mock-infected	10 hpi	10 hpi
	SFV-infected	6 hpi	

## Abbreviations

The following abbreviations are used in this manuscript:

CPV	cytopathic vacuole
EM	electron microscopy
hpi	hours post infection
MEB	midgut escape barrier
NSDV	Nairobi sheep disease virus
SFV	Semliki Forest virus
TBEV	tick-borne encephalitis virus

## References

[1] Labuda M., Nuttall P.A. (2004). Tick-borne viruses. Parasitology.

[2] Nuttall P.A. (2009). Molecular characterization of tick-virus interactions. Front. Biosci..

[3] Attoui H., Stirling J.M., Munderloh U.G., Billoir F., Brookes S.M., Burroughs J.N., de Micco P., Mertens P.P.C., de Lamballerie X. (2001). Complete sequence characterization of the genome of the St Croix River virus, a new orbivirus isolated from cells of *Ixodes scapularis*. J. Gen. Virol..

[4] Bell-Sakyi L., Attoui H. (2013). Endogenous tick viruses and modulation of tick-borne pathogen growth. Front. Cell. Infect. Microbiol..

[5] Munz E., Reimann M., Mahnel H., Yunker C.E. (1987). Nairobi sheep disease virus and *Reovirus*-like particles in the tick cell line TTC-243 from *Rhipicephalus appendiculatus*: Experiences with the handling of the tick cells, immunoperoxidase and ultrahistological studies. Arboviruses in Arthropod Cells in Vitro.

[6] Alberdi M.P., Dalby M.J., Rodriguez-Andres J., Fazakerley J.K., Kohl A., Bell-Sakyi L. (2012). Detection and identification of putative bacterial endosymbionts and endogenous viruses in tick cell lines. Ticks Tick-Borne Dis..

[7] Bell-Sakyi L., Attoui H. (2016). Virus discovery using tick cell lines. Evol. Bioinform..

[8] Katzourakis A., Gifford R.J. (2010). Endogenous viral elements in animal genomes. PLoS Genet..

[9] Lawrie C.H., Uzcategui N.Y., Gould E.A., Nuttall P.A. (2004). Ixodid and argasid tick species and West Nile virus. Emerg. Infect. Dis..

[10] Gonzalez J.P., Bouquety J.C., Lesbordes J.L., Madelon M.C., Mathiot C.C., Meunier D.M.Y., Georges A.J. (1987). Rift Valley fever virus and haemorrhagic fever in the Central African Republic. Ann. Inst. Pasteur/Virol..

[11] Konstantinov O.K. (1990). Ticks of the Ixodidae family as reservoir of arboviruses in the republic of Guinea. II. Arboviruses. Rev. Elev. Med. Vet. Pays Trop..

[12] Bell-Sakyi L., Kohl A., Bente D.A., Fazakerley J.F. (2012). Tick cell lines for study of Crimean-Congo hemorrhagic fever virus and other arboviruses. Vector-Borne Zoonot. Dis..

[13] Leake C.J., Pudney M., Varma M.G.R., Kurstak E., Maramorosch K., Dubendorfer A. (1980). Studies on arboviruses in established tick cell lines. Invertebrate Systems in Vitro.

[14] Garcia S., Billecocq A., Crance J.-M., Munderloh U.G., Garin D., Bouloy M. (2005). Nairovirus RNA sequences expressed by a Semliki Forest virus replicon induce RNA interference in tick cells. J. Virol..

[15] Garcia S., Billecocq A., Crance J.M., Prins M., Garin D., Bouloy M. (2006). Viral suppressors of RNA interference impair RNA silencing induced by a Semliki Forest virus replicon in tick cells. J. Gen. Virol..

[16] Barry G., Alberdi P., Schnettler E., Weisheit S., Kohl A., Fazakerley J.K., Bell-Sakyi L. (2013). Gene silencing in tick cell lines using small interfering or long double-stranded RNA. Exp. Appl. Acarol..

[17] Moniuszko A., Rueckert C., Alberdi M.P., Barry G., Stevenson B., Fazakerley J.K., Kohl A., Bell-Sakyi L. (2014). Coinfection of tick cell lines has variable effects on replication of intracellular bacterial and viral pathogens. Ticks Tick-Borne Dis..

[18] Siu R.W.C., Fragkoudis R., Simmonds P., Donald C.L., Chase-Topping M.E., Barry G., Attarzadeh-Yazdi G., Rodriguez-Andres J., Nash A.A., Merits A. (2011). Antiviral RNA interference responses induced by Semliki Forest virus infection of mosquito cells: Characterization, origin, and frequency-dependent functions of virus-derived small interfering RNAs. J. Virol..

[19] Fragkoudis R., Chi Y., Siu R.W.C., Barry G., Attarzadeh-Yadzi G., Merits A., Nash A.A., Fazakerley J.F., Kohl A. (2008). Semliki Forest virus strongly reduces mosquito host defence signaling. Insect Mol. Biol..

[20] Pudney M., Leake C.J., Varma M.G.R., Kurstak E. (1979). Replication of arboviruses in arthropod in vitro systems. Arctic and Tropical Arboviruses.

[21] Grimley P.M., Berezesky I.K., Friedman R.M. (1968). Cytoplasmic structures associated with an arbovirus infection: Loci of viral ribonucleic acid synthesis. J. Virol..

[22] Virtanen I., Wartiovaara J. (1974). Virus-induced cytoplasmic membrane structures associated with Semliki Forest virus infection studied by the freeze-etching method. J. Virol..

[23] Hellstrom K., Vihinen H., Kallio K., Jokilato E., Ahola T. (2015). Correlative light and electron microscopy enables viral replication studies at the ultrastructural level. Methods.

[24] Senigl F., Kazimirova M., Labuda M., Nuttall P.A. (2000). Maturation of tick-borne encephalitis virus in tick and mammalian cells. *Ticks and Tick-borne Pathogens: Into the 21st Century*, Proceedings of the 3rd International Conference on Ticks and Tick-borne Pathogens.

[25] Senigl F., Kopecky J., Grubhoffer L. (2004). Distribution of E and NS1 proteins of TBE virus in mammalian and tick cells. Folia Microbiol..

[26] Martin H.M., Vidler B.O. (1962). In vitro growth of tick tissues (*Rhipicephalus appendiculatus* Neumann, 1901). Exp. Parasitol..

[27] Yunker C.E., Cory J. (1967). Growth of Colorado tick fever virus in primary tissue cultures of its vector, *Dermacentor andersoni* Stiles (Acarina: Ixodidae), with notes on tick tissue culture. Exp. Parasitol..

[28] Chunikhin S.P., Khozinskaya G.A., Stefutkina L.F., Korolev M.B. (1984). Mono- and mixed infections of tissue explants of ticks of the genus *Hyalomma* with tick-borne encephalitis and Powassan viruses. Parazitologiya.

[29] Khozinskaya G.A., Chunikhin S.P., Khozinsky V.V., Stefutkina L.F. (1985). Variability of Powassan virus cultured in tissue explants and organism of *Hyalomma anatolicum* ticks. Acta Virol..

[30] Bell L.J., Griffiths D.A., Bowman C.E. (1984). Tick tissue culture techniques in the study of arthropod-borne protozoa: The development of *Theileria annulata* in organ cultures of *Hyalomma anatolicum anatolicum*. Acarology 6.

[31] Fragkoudis R., Breakwell L., McKimmie C., Boyd A., Barry G., Kohl A., Merits A., Fazakerley J.K. (2007). The type I interferon system protects mice from Semliki Forest virus by preventing widespread virus dissemination in extraneural tissues, but does not mediate the restricted replication of avirulent virus in central nervous system neurons. J. Gen. Virol..

[32] Tamberg N., Lulla V., Fragkoudis R., Lulla A., Fazakerley J.K., Merits A. (2007). Insertion of EGFP into the replicase gene of Semliki Forest virus results in a novel, genetically stable marker virus. J. Gen. Virol..

[33] Jose J., Tang J., Taylor A.B., Baker T.S., Kuhn R.J. (2015). Fluorescent protein-tagged Sindbis virus E2 glycoprotein allows single particle analysis of virus budding from live cells. Viruses.

[34] Bell-Sakyi L. (2004). *Ehrlichia ruminantium* grows in cell lines from four ixodid tick genera. J. Comp. Pathol..

[35] Igarashi A. (1978). Isolation of a Singh’s *Aedes albopictus* cell clone sensitive to Dengue and Chikungunya viruses. J. Gen. Virol..

[36] Attarzadeh-Yazdi G., Fragkoudis R., Chi Y., Siu R.W., Ülper L., Barry G., Rodriguez J.A., Nash A.A., Bouloy M., Merits A. (2009). Cell-to-cell spread of the RNAi response suppresses Semliki Forest virus infection of mosquito cell cultures and cannot be antagonized by this virus. J. Virol..

[37] Macpherson I. (1963). Characteristics of a hamster cell clone transformed by polyoma virus. J. Natl. Cancer Inst..

[38] Froshauer S., Kartenbeck J., Helenius A. (1988). Alphavirus RNA replicase is located on the cytoplasmic surface of endosomes and lysosomes. J. Cell Biol..

[39] Frolova E.I., Gorchakov R., Pereboeva L., Atasheva S., Frolov I. (2010). Functional Sindbis virus replicative complexes are formed at the plasma membrane. J. Virol..

[40] Bell-Sakyi L., Palomar A.M., Bradford E.L., Shkap V. (2015). Propagation of the Israeli vaccine strain of *Anaplasma centrale* in tick cell lines. Vet. Microbiol..

[41] Hayasaki D., Yoshii K., Ueki T., Iwasaki T., Takashima I. (2004). Sub-genomic replicons of *Tick-borne encephalitis virus*. Arch. Virol..

[42] Gehrke R., Heinz F.X., Davis N.L., Mandl C.W. (2005). Heterologous gene expression by infectious and replicon vectors derived from tick-borne encephalitis virus and direct comparison of this flavivirus system with an alphavirus replicon. J. Gen. Virol..

[43] Booth T.F., Gould E.A., Nuttall P.A. (1991). Structure and morphogenesis of Dugbe virus (Bunyaviridae, Nairovirus) studied by immunogold electron microscopy of ultrathin cryosections. Virus Res..

[44] Offerdahl D.K., Dorward D.W., Hansen B.T., Bloom M.E. (2012). A three-dimensional comparison of tick-borne flavivirus infection in mammalian and tick cell lines. PLoS ONE.

[45] Gresíková M., Sekeyová M., Tempera G., Guglielmino S., Castro A. (1978). Identification of a Sindbis virus strain isolated from *Hyalomma marginatum* ticks in Italy. Acta Virol..

[46] Lwande O.W., Lutomiah J., Obanda V., Gakuya F., Mutisya J., Mulwa F., Michuki G., Chepkorir E., Fischer A., Venter M. (2013). Isolation of tick and mosquito-borne arboviruses from ticks sampled from livestock and wild animal hosts in Ijara District, Kenya. Vector-Borne Zoonot. Dis..

[47] Linthicum K.J., Logan T.M., Bailey C.L., Gordon S.W., Peters C.J., Monath T.P., Osorio J., Francy D.B., McLean R.G., Leduc J.W. (1991). Venezuelan equine encephalomyelitis virus infection in and transmission by the tick *Amblyomma cajennense* (Arachnida: Ixodidae). J. Med. Entomol..

[48] Linthicum K.J., Logan T.M. (1994). Laboratory transmission of Venezuelan equine encephalitis virus by the tick *Hyalomma truncatum*. Trans. R. Soc. Trop. Med. Hyg..

